# Investigating the Levels of Serum Vitamin D in Patients with Rheumatoid Arthritis Referred To Rasoul-Akram Hospital During 2011-2012

**Published:** 2014-09

**Authors:** Mahdi Fakharan, Anousheh Haghighi, Mohsen Arabi, Maryam Loghman

**Affiliations:** 1 Internal Medicine Trainee, Department of Internal Medicine, Rasoul-Akram Hospital, Iran University of Medical Sciences, Tehran, Iran;; 2 Rheumatologist, Department of Internal Medicine, Rasoul-Akram Hospital, Iran University of Medical Sciences, Tehran, Iran

**Keywords:** Vitamin D, Rheumatoid arthritis, Iran

## Abstract

Vitamin D_3_ has a role in many autoimmune diseases and appears to play a function in controlling Rheumatoid Arthritis (RA). The aim of this study is to evaluate the relationship between serum level of vitamin D and RA disease activity score. The serum level of vitamin D in 75 RA patients referred to the rheumatology clinic of Rasoul-Akram hospital was measured. Patients were classified into low, moderate and high RA activity groups based on the DAS-28 criteria (Disease Activity Score in 28 joints) and the mean values of serum vitamin D were compared between the three groups.

The mean serum levels of vitamin D in high activity group (17.057±7.7 mg/ml) was significantly less than moderate (30.5±11.3 mg/ml) and low (36.7±19.5 mg/ml) activity groups (P<0.001). The outcome of this study shows that serum level of vitamin D is inversely correlated with the activity of RA.

## Introduction

Rheumatoid arthritis (RA) is the most common inflammatory arthritis that affects about 0.5% to 1% of the world population.^1^ The etiology of rheumatoid arthritis is not well understood, but it has been shown that both environmental and genetic factors are important in the distribution and prevalence of the disease.^1^

Vitamin D_3_ has a role in many autoimmune diseases and appear to play a function in controlling RA. The discovery of vitamin D receptors on immune cells as well as the finding that vitamin D has some effects on immune cells, suggests that vitamin D_3_ may have a regulatory action on the immune system.^2^^,^^3^ Studies have shown that both direct and indirect immunomodulating effects of vitamin D on T cells, B cells and antigen-presenting cells (dendritic cells and macrophages).^2^ This may explain the stated association between vitamin D deficiency and some types of autoimmune diseases. It is possible that administration of vitamin D in patients with RA can help suppressing adverse reactions of immune system activity. The level of serum vitamin D depends on several factors, such as the amount of sun exposure, genetic background (vitamin D receptor polymorphism) and diet.

Different investigations have shown contradicting evidences on the relationship between the serum levels of vitamin D and the RA activity rate.^4^^-^^11^ While several studies show an inverse correlation,^7^^,^^8^ others fail to provide any association between vitamin D and RA activity.^9^^,^^11^

This cross-sectional study aims at determining the serum levels of vitamin D_3_ in patients with rheumatoid arthritis. This would provide a better understanding of the relationship between the levels of serum vitamin D and RA among the Iranian population. It also provides insight towards etiology, prevalence and prevention of RA.

## Materials and Methods

This cross-sectional study was performed at Rasoul-Akram hospital, Tehran, Iran during 2011-2012. According to previous studies and based on comparing two means formula, 300 consecutive patients diagnosed with rheumatoid arthritis (RA) in-line with criteria by the American College of Rheumatology (ACR) were included. DAS-28 was used to measure RA activity and patients were categorized accordingly into three groups. 86 patients were classified in the low activity (DAS28<3.2), 152 patients in the moderate activity (3.2<DAS28<5.1) and 62 patients in the high activity (DAS28>5.1) groups. Exclusion criteria was: (i) patients having had any form of vitamin D supplementations (except for calcium-D pills which contains 200 IU of vitamin D) during previous three months, (ii) patients with history of parathyroid disease and hyperthyroidism, kidney or liver diseases and diabetes, (iii) pregnant females. Following demographic characterization, 75 patients with rheumatoid were considered for the investigation and were placed in three groups of 25 individuals.

The serum 25-OH vitamin D levels and erythrocyte sedimentation rate (ESR) were obtained for all patients during their routine laboratory analysis. The levels of the serum 25-OH vitamin D were determined using Electrochemiluminescence method (EUROIMMUN GmbH). Biochemical and demographic data, including gender and age were collected and recorded. The serum levels of vitamin D higher than 30ng/ml was considered as normal, between 20 and 30ng/ml as insufficient and below 20ng/ml as deficiency. Quantitative and qualitative variables were analyzed using independent *t-* test, one-way ANOVA, post hoc tukey test and chi-square (SPSS v. 16.0) respectively. P>0.005 were considered as significant.

There was neither conflict of interest nor any financial gain from the results of this study.

## Results

In this study, 75 patients with RA were investigated including 9 (12%) men and 66 (88%) women. Patients were classified into low, moderate and high activity groups (each 25 patients) based on DAS28 criteria. The average age of patients was not significantly different between the groups (P=0.59, table 1) and gender as well as disease duration were comparable among groups.

**Table 1:** Comparison of age, Tender and Swellen joint, ESR, CRP and serum levels of vitamin D between groups

**Parameter mean±SD**	**Group**			**P value**
	**Low activity**	**Moderate activity**	**High activity**	
Age “year”	49.5±10.7	51.7±14.5	52.8±9	0.5
Swollen joint count	0.64±0.31	3.4±2.9	4±2.6	<0.001
Tender joint count	1.4±0.7	4.7±2.1	4.9±2.8	0.001
ESR*	16.5±9.1	20.4±12.1	27.4±17.3	0.04
CRP**	0.56±0.21	1.5±1.1	2.8±1.9	0.03
Vit D*** (ng/ml)	36.7±19.5	30.5±77.3	17.06±7.7	<0.001

*Erythrocyte Sedimentation Rate; **C. Reactive Protein; ***Vitamin D

Tender joint count and swollen joint count was significantly different between groups. Mean tender joint count in the low activity group, moderate activity group and high activity group were 1.7±1.4, 4.7±3.6 and 5.2±5.1 respectively. This was significantly different between the first group and the other two groups (P=0.003 and P=0.002). Mean swollen joint count in the low activity group, moderate activity group and high activity group were 0.86±0.84, 4±2.9 and 5.04±4.7 respectively. Yet again, there was statistically significant difference between the first group and the other two groups (P<0.001 and P<0.001).The average levels of serum vitamin D was found to be statistically different (P<0.001, table 1).

In the group with high RA activity, the level of serum vitamin D was lower (7.05±7.7mg/ml) than the other two groups (table 1). Paired comparison of serum vitamin D levels between groups shows statistically significant difference between the low and the high activity groups (P<0.001). This difference was also significant between the moderate and the high activity groups (P=0.003).

The serum levels of vitamin D was found to be inversely related to ESR (P<001, r=-0.8).

## Discussion

Although the role of vitamin D_3_ is established in many autoimmune diseases and it appears to play a function in controlling RA, different investigations have shown contradicting evidences on the relationship between the serum levels of vitamin D and the RA activity rate. The current study aimed at determining the relationship between the levels of serum vitamin D and RA activity.

Patients were classified into three groups of low, moderate and high RA activity. The average age, gender and disease duration in all groups were similar and thus, there was no confounder in this study.

In the current study, the average serum levels of vitamin D in the group with high activity was significantly less than the moderate and the low activity groups (P<0.001). 

Previous studies on this topic have been controversial. In a study by Turhanglu (2011) using DAS28 criteria, a reverse association between RA activity and serum levels of vitamin D in 65 RA patients was shown. However, contrary to other studies, they found that serum vitamin D levels in patients with RA were similar to those in the healthy controls.^7^ In a similar study by Haque and colleagues (2010), serum vitamin D level was correlated inversely with pain, DAS score and disability level.^4^ In another study on 266 patients, Steven (2009) also showed a reverse correlation with the levels of serum vitamin D and RA activity. In that study, it was shown that patient with high RA activity had a deficiency in their vitamin D levels.^6^ In 2012, Ifigenia Kostoglou-Athanassiou et al. measured the level of vitamin D in a cohort of 44 RA patients to assess the relationship between serum level of vitamin D and disease activity. They showed that vitamin D deficiency is highly prevalent in patients with RA, and that vitamin D deficiency may be linked to disease severity in RA.^12^ Cutolo et al. found that plasma levels of vitamin D were inversely correlated with the RA disease activity showing a circannual rhythm (more severe in winter).^13^

Contrary to some previous studies, Baker (2012) showed that serum level of vitamin D is not correlated with RA activity and response to treatment was similar in patients with different levels of vitamin D.^10^ In contrast to other investigations, all patients taking part in Baker’s study had an active disease and were candidates for treatment with biological drugs. This may be the cause of difference between the results of this study and previous investigations.

It has been shown that the intake of vitamin D can reduce RA incidence in middle-aged females. In an investigation on 29,368 females (55-69 years old) over a period of eleven years follow-up, Merlino^5^ showed that those who received vitamin D supplement had lower RA rate. It was further demonstrated that serum levels of vitamin D in patients with ESR>24 was significantly lower than those with ESR<24. This suggests an anti-inflammatory effect of vitamin D besides its anti-osteoporosis effect, by which it can alleviate the RA symptoms.

Thus, similar to most past investigations, this study also shows significant relationship between the serum levels of vitamin D and RA activity. However, due to the small number of patients used in the current study, further investigation with more patients is required for a better understanding of the correlation between RA activity and vitamin D. 

## Conclusion

This study shows that patients with high RA activity have significantly lower level of serum vitamin D compared with patients with moderate or low RA activity. It is concluded that administration of vitamin D may reduce RA activity. Performing an interventional study on two RA groups with greater number of patients receiving supplementary vitamin D or placebo, may provide better insights towards understanding the correlation between vitamin D and rheumatoid arthritis pathogenesis.
